# Development of evaluation index system for functional ability of older patients with stroke based on healthy aging: a modified Delphi study

**DOI:** 10.3389/fpubh.2025.1562429

**Published:** 2025-03-13

**Authors:** Wei Zhang, Xiu-bin Tao, Xiao-li Fan, Ai-ping Wang

**Affiliations:** ^1^The First Affiliated Hospital of China Medical University, Shenyang, China; ^2^The First Affiliated Hospital of Wannan Medical College, Wuhu, China; ^3^Key Laboratory of Public Health Social Governance, Philosophy and Social Sciences of Anhui Province, Hefei, China

**Keywords:** functional ability, older individuals, stroke, Delphi study, index system

## Abstract

**Background:**

The prevalence of stroke in the older population is high and it is critical to promote and maintain the functional status of older patients post stroke. Health measures centered on functional ability can scientifically reflect the health status of older individuals. The aim of this study was to develop an evaluation index system for assessing the functional ability of older patients with stroke based on the World Health Organization Healthy Aging Model.

**Methods:**

Key indicators were identified through literature analysis and semi-structured interviews with 10 older patients with stroke. A two-round expert consultation process was conducted to evaluate and revise the indicators. Subsequently, a hierarchical construction model was established using the analytic hierarchy process to determine the weight of each level indicator.

**Results:**

The evaluation index system comprised three first-level, 13 s-level, and 53 third-level indicators. The weights ranged from 0.143–0.429 for first-level indicators, 0.052–0.349 for second-level indicators, and 0.040–0.667 for third-level indicators.

**Conclusion:**

The developed evaluation index system demonstrates reliability for assessing the functional ability of older stroke patients and provides a standardized framework for nursing staff to conduct functional assessment of older stroke patients.

## Introduction

The global older population has experienced continuous growth since the beginning of the 21st century. World Bank data showed that the world’s population aged 65 and above has increased to approximately 779 million by 2022, and it is expected that by 2030, the global older population will account for about 22% of the total population (1). Data from China’s 7th National Population Census reveal that the population aged 60 and above has surpassed 267 million, representing approximately 18.8% of the total population (2). While the older population growth is accelerating, the health outcomes among the older population in China remain concerning. Multi-morbidity and chronic disease prevalence are particularly high in this demographic (3). Studies indicate that 88.6% of older adults suffer from at least two chronic disease (4), with stroke being particularly prevalent among those aged 65 years, which is the most common group of patients suffering from stroke in China (5).

Globally, stroke ranks as the second leading cause of death and disability, and the direct medical costs and indirect productivity losses caused by stroke disability have become an important public health challenge in aging societies (6). Moreover, older patients demonstrate increased vulnerability to post-stroke dysfunctions compared with younger patients, owing to age-related organ function decline. These include varying degrees of neurological deficits (7), post-stroke cognitive impairment (8), psychological dysfunction (9), and limitations in performing daily activities, potentially impeding full health recovery. Therefore, strengthening the functional assessment and early rehabilitation of older adult stroke patients, improving the community support system and promoting preventive health education are key measures to improve public health.

The WHO proposed the Global Report on Ageing and Health in 2015, which increased the concept of healthy aging, emphasizing mobility and social functions in older individuals’ health status. The report further elaborated on it as a necessary process of the functional ability for developing and maintaining a healthy life for older individuals (10). The revised concept of healthy aging defines functional ability as the product of the interaction between an individual’s intrinsic capacity and environmental factors (11). The WHO framework integrates the previous physical and mental health functions into the intrinsic capacity of older individuals and proposes that the five dimensions of “activity, vitality, sensory, cognitive and psychological” should be considered as a whole to comprehensively assess the intrinsic capability of older individuals, which can be used to rapidly screen and assess the functional status (12). Besides, environment factors are incorporated to the connotation of healthy aging, including micro-level personal living environment, family environment factors, and macro level building an aging friendly society environmental factors.

Regarding the health of stroke patients from the perspective of functional ability, the focus is on the patient’s intrinsic capability and characteristics of the surrounding environment. Impaired intrinsic capacity in stroke patients leads to a direct reduction in their level of functional ability. Firstly, the impairment of intrinsic capacity is directly related to stroke-induced dysfunction, which varies based on type of stroke and lesion area; for example, injury to the middle or posterior cerebral artery causes sensory, language and cognitive deficits that affect the subjective expression of patients; lesions of the internal capsule cause visual deficits; and lesions of the basal ganglia region may cause hemiplegia, affecting hand function and walking, resulting in a lack of mobility for the patient, failing to ensure their basic quality of life. Secondly, a series of complications caused by stroke, such as post-stroke dysphagia, choking on drinking water, and post-stroke depression (13), indirectly led to a decline in intrinsic capacity. Poor oral function may cause dysphagia, which can directly affect the intake of nutrients in older patients, leading to malnutrition and low immunity, causing disorders in energy metabolism and decreasing their intrinsic capacity (14). In addition, some stroke patients worry that they will add to the burden on their families, indirectly resulting in symptoms such as low mood and reluctance to express themselves, which affect their cognitive functioning and ability to live, leading to a decline in the quality of their survival (15).

However, intrinsic capacity is only one of the factors determining what the patient can do, and functional ability is also affected by the strengths and weaknesses of the resources in the environment (10). For example, whether older patients with stroke who have limited physical mobility can use public transportation facilities with the aid of assistive devices, and whether older patients with stroke who walk normally have the opportunity to participate in community activities. This suggests that patients are most likely to establish and maintain optimal functional abilities when they adapt well to the environment. Therefore, it is necessary to comprehensively assess the intrinsic capacity and environmental factors of older patients with stroke, explore the factors influencing their functional ability, and provide targeted interventions to effectively maintain the intrinsic capacity of older patients with stroke to improve their functional ability. However, because the basis for evaluating the functional ability of older patients with stroke remains unclear, this study was based on the latest WHO definition of healthy aging and believes that the degree of functional ability of older patients with stroke depends on their intrinsic capacity, environmental support, and the interaction between the two. To develop a system of evaluation indices for the functional ability of older patients with stroke in terms of these three dimensions, to provide a reference to clinical practice, which has important and far-reaching significance for reducing the recurrence, disability, and mortality rates of cerebrovascular disease in the older individuals and improving the quality of life and prognosis of older patients with stroke.

## Materials and methods

### Study design

This is a modified Delphi study employing an integrated design method. A team was established to conduct this study, including one nurse manager from each of the neurology, neurosurgery, and rehabilitation departments and two master’s degree nursing students. First, we combined a literature review and semi-structured interviews to develop a preliminary draft of evaluation indicators for the functional ability of older patients with stroke. Second, an iterative two-round Delphi survey is conducted to develop an evaluation index system. A Delphi study can effectively evaluate the problem details. This is primarily because the Delphi method is a multistage iterative process; therefore, relevant experts can consider each other’s opinions and reach a consensus. This study was conducted following the Guidelines for Conducting and Reporting Delphi Studies (16).

### Literature analysis

Relevant studies were analyzed to determine the keywords for the literature search and expand the search terms based on the retrieved articles. The main keywords including “older adult,” “aged,” “old,” “geriatrics,” “healthy aging,” “stroke,” “functional ability and “intrinsic capacity” in five international databases (PubMed, CINAHL, PsycINFO, Elsevier ScienceDirect and Wiley Online Library) and three domestic (Chinese) databases (China National Knowledge Infrastructure, Wan Fang databases and VIP) from the database’s inception to May 2023. The search strategies are presented in Supplementary file 1.

The inclusion criteria were as follows: (a) available full text of the literature and (b) relevant to the current study, including guidelines, systematic reviews, meta-analyses, original studies, and evidence summaries. The exclusion criteria were as follows: (a) duplicate studies and (b) flawed study design.

A total of 2,619 relevant literature were assessed, and 49 key articles were analyzed. The following three main dimensions and corresponding themes were extracted from the literature analysis: intrinsic capacity (activity function, visual ability, cognitive function, psychological state, and vitality state), external environment (social support, social security, public health services, family function, economic level, and living environment), and individual-environment interaction (social participation), including three first-level indicators, 16 s-level indicators, and 51 third-level indicators.

### Semi-structured interviews

In this study, older patients with stroke were selected using a purposive sampling method from the Department of Neurology, Neurosurgery, Rehabilitation at a tertiary hospital in China during 1 September 2023 and 31 October 2023 for face-to-face semi-structured interviews. The interviews aimed to obtain the perceptions of older patients with stroke regarding their ability to meet their basic needs, thereby supplementing the evaluation indicators. The inclusion criteria for patients: (a) 60 years old and above; (b) No cognitive impairment; (c) Previous history of stroke; (d) Interested in interviews and volunteer participation. Exclusion criteria: (a) patients with speech comprehension disorders or severe auditory or visual impairments and (b) inability to cooperate due to an unstable condition, weakness, inability to tolerate, or previous history of mental illness. All the participants agreed to be interviewed and signed a written informed consent form.

Interview topics were set according to the International Classification of Functioning, Disability, and Health (ICF) frameworks. ICF is a standardized framework released by WHO for describing individual health and functional status in different health fields, which is based on the concept of biological, psychological, and social health and provides a standardized collection, evaluation, and analysis method for evaluating the functional status of patients (17). Through literature review and consultation with experts, preliminary interview questions were developed, and two patients were pre-interviewed. Based on the pre-interview results, the interview questions were revised and improved, and the final interview questions were determined for the formal interviews. The interview guide is provided in Supplementary file 2.

The interviews were conducted anonymously with the promise that the information obtained would be used only for academic studies. A trained researcher organized each interview which lasted for 20–30 min. The interview was terminated when there was no new topic information and the data appeared repeatedly during the interview. Ultimately, ten patients were included in this study. After each interview, recordings were converted into verbatim text. The recordings of each interviewee were numbered and archival data were created.

Thematic analysis was used to analyze the interview data. The first step involved reading the transcribed text and reflective records. The second step was to analyze the transcribed text sentence-by-sentence to identify significant statements and code them based on the four dimensions of the ICF. Next, coding was organized to form potential themes, followed by the collection of all relevant information for each potential theme. Finally, all codes were classified and themes were defined and named based on the information under the code. This cycle was continued until no new themes appeared.

Finally, evaluation indicators of the functional ability of older patients with stroke were identified, including three first-level indicators, 17 s-level indicators, and 57 third-level indicators.

### Develop an expert inquiry questionnaire

The expert inquiry questionnaire was designed based on the preliminary evaluation indicators for the functional ability of older patients with stroke, which included five parts: (a) Questionnaire description: plain language summaries of background and purpose of the study, filling requirements, and response time requirements to make the survey more accessible; (b) Questionnaire text: items of the evaluation indicators, and experts evaluated the importance of these indicators based on a Likert 5-point rating scale, with a modified opinion column attached. Experts can provide suggestions and reasons for the modification, addition, and deletion of items; (c) a questionnaire of expert basic information, including name, sex, age, study field, education, work unit, position, and professional title; and (d) Likert rating scale used to evaluate experts’ familiarity with the question from 0 to 1 (1 = very familiar, 0.75 = more familiar, 0.5 = generally familiar, 0.25 = less familiar, 0 = unfamiliar). The experts’ judgment basis was assessed through judgment scores, which represent the degree of influence from 0 to 1 (0 = small, 0.5 = medium, 1 = large) based on practical experience (0.4), theoretical analysis (0.3), understanding of peers (0.2), and intuitive choice (0.1). Both of the above scoring methods are common in conducting Delphi surveys, are reliable and valid, and were not designed in this study but were selected concerning previous studies. The expert inquiry questionnaire and evaluation criteria (18) are shown in Supplementary file 3.

### Selection of experts

Experts nationwide in China were selected to participate in the Delphi survey, and the inclusion criteria for experts were as follows: (a) working unit: tertiary comprehensive hospital or higher education institution; (b) study fields: chronic disease management or aging care and have been engaged in this study field for 10 years or more; (c) educational level: bachelor’s degree and above; (d) professional title: associate senior or above; and (e) voluntary participation, and can ensure continuous participation in several rounds of the Delphi survey.

### Implementation of the Delphi survey

A Delphi survey was conducted between March and May 2023. After verbal consent was obtained from the experts, questionnaires were distributed via email. If the experts failed to respond promptly, an email reminder was sent. In each round of the Delphi survey, we repeated the invitation to the experts who participated in the previous round of the Delphi survey to encourage expert consultation. During the Delphi survey, experts emailed their comments to us. The research team revised the indicators in response to the experts’ comments. First, data from the first round of the Delphi survey were analyzed to include and exclude indicators. The inclusion criteria for indicators were: mean importance score ≥ 3.5, coefficient of variation ≤0.25, and rate of expert recognition >70% (16); Secondly, modifications were made to the issues of unclear expression, overlapping indicators, and mutual inclusion in the first round of the Delphi survey, and indicators that met the inclusion criteria were included in the next round Delphi survey. If expert opinions on the indicator were controversial, the study group discussed them and decided whether the indicator should be deleted or retained. The above steps were repeated until there was a convergence of expert opinions, and then the Delphi survey was stopped. This study aimed to develop a system of evaluation indicators, the application of which will be further developed in a subsequent study and is therefore not covered in this study.

### Statistical analysis

SPSS software (version 22.0) was used to analyze the data. Descriptive analyses were performed using the mean values, standard deviations, coefficients of variation, and proportions. The validity of the Delphi expert consultation was demonstrated by the positive, authority (Cr), and coordination coefficients. The positive coefficient of the experts was expressed by the recovery rate of the effective questionnaires; a recovery rate greater than 70% was considered good (19). The judgment coefficient (Ca) represents evidence when an expert makes a judgment, whereas the familiarity coefficient (Cs) represents the degree of the expert’s familiarity with the problem (20). The arithmetic average of Ca and Cs is used to represent the Cr of experts, that is Cr = (Ca + Cs) / 2; with an acceptable range of Cr ≥ 0.7 (20).

The degree of expert coordination (21) is an important index for judging the consistency of indicators among experts and is represented by each indicator’s coefficient of variation (Cv) and Kendall’s W coordination coefficient. Cv reflects the relative dispersion of the experts’ scores on the evaluation of the importance of the indicator, which is a basis for deletion of the indicator (20). Kendall’s W coefficient reflects the consistency of the experts’ evaluation results for the indicator’s importance. The larger the Kendall’s W coefficient, the higher the degree of expert coordination and consistency of expert opinion.

## Results

### Interview findings

Five themes and seven sub-themes were identified by analyzing the interview data. According to the information of themes, one new second-level indicator, that is “traffic conditions,” and six new third-level indicators were incorporated, including “hemianesthesia,” “positive emotions,” “communication skills,” “housework,” “transportation facilities,” “traffic safety” (See Table 1).

**Table 1:** Themes and sub-themes identified through analysis of semi-structured interviews.

Categories (ICF)	Themes	Sub-themes	Examples of meaning units (quotes)
Body functions	1 Sensory function and pain	1.1 Numbness or sensory loss	“It’s just numbness in the right hand now, and it’s usually difficult to hold chopsticks for meals.” (P5, participant)
			“It was fine when I was first discharged from the hospital, but recently I started to have numbness in my feet, and lifting my feet is affected.” (P6, participant)
		1.2 Single-site or generalized pain	“I’ve recently started to get a bit of a headache and pain in my legs so I’ve come back to the hospital for a check-up.” (P2, participant)
			“I get headaches at home sometimes and I feel uncomfortable everywhere in my body.” (P5, participant)
	2 Emotional function	2.1 Fear of disease outcome	“I felt so slow to recover and worried that I was getting worse, sometimes I could not sleep at night and had to take sleeping pills.” (P2, participant)
			“I would just worry about how my treatment was going to be and worry that it would not work.” (P4, participant)
		2.2 Discrimination	“Because of the illness sometimes I get dizzy and walk unsteadily, but I do not want to be on crutches either, it’s ugly.” (P1, participant)
Activities and participation	1 Communication function	1.1 Communication difficulty	“It used to be fine to talk to others on the phone, now sometimes I do not even want to talk.” (P2, participant)
	2 Family life skills	2.1 Insufficient family living capacity	“I basically do not do anything at home now, my partner does all the housework.” (P4, participant)
Environmental factors	1 Utility services and policies	1.1 Transport constraints	“The traffic here is chaotic and prone to traffic accidents that I usually do not dare to ride my motorbike. “(P5, participant)
			“I live far away from here, and it’s not convenient to take the bus every time I go back and forth. The travelling expenses are so much money.” (P9, participant)

### The demographics of experts

This study invited 20 experts from tertiary hospitals and medical universities across nine provinces (cities) of China, including Beijing, Guangdong Province, Shandong Province, Jiangxi Province, Anhui Province, Jiangsu Province, Jilin Province, Hubei Province, and Fujian Province, as well as medical higher education institutions. Demographic information of the experts is presented in Table 2.

**Table 2:** Demographics data of experts.

Characteristics	Categories	Round 1 *n* (%)	Round 2 *n* (%)
Questionnaire response	Distribute / recover	20/18 (90.0)	18/15 (83.3)
Gender	Female	18 (100)	15 (100)
Age (years)	30–40	2 (11.1)	2 (13.3)
	41–50	9 (50.0)	7 (46.7)
	≥51	7 (38.9)	6 (40.0)
Occupation	Physician	3 (16.7)	3 (20.0)
	Nurses	15 (83.3)	12 (80.0)
Education	Bachelor’s degree	4 (22.2)	4(26.7)
	Master’s degree	9 (50.0)	7 (46.7)
	Doctor’s degree	5 (27.8)	4 (26.7)
Profession titles	Associate senior	9 (50.0)	8 (53.3)
	Senior	9 (50.0)	7 (46.7)
Professional experience (years)	10–20	4 (22.2)	2 (13.3)
	21–30	8 (44.4)	8 (53.3)
	≥31	6 (33.3)	5 (33.3)
Professional field (multiple choices)	Aging care	10 (55.6)	7 (46.7)
	Chronic disease management	9 (50.0)	9 (60.0)
Mentor type	Master’s supervisor	11 (61.1)	8 (53.3)
	Doctoral supervisor	2 (11.1)	2 (13.3)

### Expert positive coefficient

Two rounds of expert consultations were conducted. In the first round, 20 questionnaires were distributed and 18 questionnaires were collected, with an effective response rate of 90.0%. In the second round, 18 questionnaires were distributed and 15 were collected, with an effective response rate of 83.3%. In the first round of expert consultation, 11 experts (61.1%) proposed 35 suggestions, and in the second round of expert consultation, six experts (40.0%) proposed 17 suggestions.

### Expert authority coefficient

The Cs of an expert of two rounds were 0.75 and 0.79, the Ca of an expert were 0.93 and 0.94, and the Cr of an expert were 0.84 and 0.86, respectively. The expert authority coefficient for the two rounds of expert consultations was greater than 0.7.

### The degree of expert coordination

Kendall’s W coefficient for the first round of expert consultation was 0.238–0.278 (*p* < 0.05), and Cv was 0–0.38; Kendall’s W coefficient for the second round of expert consultation was 0.350–0.406 (*p* < 0.05), and Cv was 0–0.287, as shown in Table 3. Based on the statistical results of each indicator after second round of expert consultation, Kendall’s W coefficient increases, and there is a convergence of opinions among experts; therefore, the next round of expert consultation was not conducted.

**Table 3:** Expert coordination coefficients.

Categories	Indicators	Items	Kendall’s W	Chi-square	Degree of freedom	*p* values
First round	First-level indicators	3	0.278	10.000	2	0.007
	Second-level indicators	17	0.238	68.471	16	0.000
	Third-level indicators	57	0.264	266.003	56	0.000
	All indicators	77	0.254	347.341	76	0.000
Second round	First-level indicators	3	0.406	12.182	2	0.002
	Second-level indicators	13	0.391	70.313	12	0.000
	Third-level indicators	53	0.350	273.143	52	0.000
	All indicators	69	0.453	462.161	68	0.000

### Results of the first round of the Delphi survey

Thirty-one comments were received after the first round of expert consultation, totaling 35 comments. We integrated the comments and revised the indicators in combination with the inclusion criteria. One second-level and seven third-level indicators were added, and the two second-level indicators were merged. Experts suggested that the indicators of “interpersonal relationships” and “social support” intersect and should be merged; Experts suggested that “housing environment” and “transport condition” belong to “living environment,” so we incorporated all third-level indicators included in that two secondary indicators into “living environment”; One second-level indicator and eight third-level indicators were modified; Two second-level indicators and ten third-level indicators were excluded. After modifying the indicators, a second round of expert consultations was conducted.

### Results of the second round of the Delphi survey

Seventeen comments were received after the second round of expert consultations. One second-level indicator and four third-level indicators were excluded, one second-level indicator and seven third-level indicators were added, and four third-level indicators were revised. Finally, an evaluation index system for the functional ability of older patients with stroke was determined, including three first-level indicators, 13 s-level indicators, and 53 third-level indicators (See Table 4). The results of the indicator revisions are shown in Supplementary file 4.

**Table 4:** Weight and consistency test results of each level indicator.

Indicators	Importance score (¯*x* ± *s*)	Cv	Weight
1 Intrinsic capacity	5.00 ± 0.00	0	0.429
1.1 Activity function	4.87 ± 0.52	0.106	0.255
1.1.1 Basic daily activity ability	4.93 ± 0.26	0.052	0.667
1.1.2 Instrumental daily activity ability	4.80 ± 0.41	0.086	0.333
1.2 Sensory function	4.73 ± 0.46	0.097	0.144
1.2.1 Visual ability	4.93 ± 0.26	0.052	0.222
1.2.2 Hearing ability	4.87 ± 0.35	0.072	0.122
1.2.3 Deep and shallow sensory ability of the body	4.73 ± 0.59	0.125	0.111
1.2.4 Gustatory ability	4.93 ± 0.26	0.052	0.222
1.2.5 Olfactory ability	4.93 ± 0.26	0.052	0.222
1.3 Cognitive function	4.80 ± 0.41	0.086	0.169
1.3.1 Memory	4.87 ± 0.35	0.072	0.182
1.3.2 Attention	4.80 ± 0.56	0.117	0.145
1.3.3 Calculation ability	4.93 ± 0.26	0.052	0.230
1.3.4 Directive force	4.87 ± 0.52	0.106	0.182
1.3.5 Language ability	4.73 ± 0.59	0.125	0.115
1.3.6 Visual spatial discrimination ability	4.80 ± 0.56	0.117	0.145
1.4 Psychological state	4.93 ± 0.26	0.052	0.288
1.4.1 Post-stroke comorbid anxiety and depression	4.73 ± 0.46	0.097	0.128
1.4.2 Positive emotions	4.93 ± 0.26	0.052	0.280
1.4.3 Psychological resilience	4.93 ± 0.26	0.052	0.280
1.4.4 Self-efficacy	5.00 ± 0.00	0	0.312
1.5 Vitality state	4.73 ± 0.59	0.125	0.144
1.5.1 Nutritional state	4.93 ± 0.26	0.052	0.289
1.5.2 Sleep state	4.80 ± 0.41	0.086	0.176
1.5.3 Chronic pain	4.93 ± 0.26	0.052	0.289
1.5.4 Vascular health state	4.87 ± 0.35	0.072	0.247
2 External environment	4.67 ± 0.49	0.105	0.142
2.1 Social support	4.73 ± 0.59	0.125	0.192
2.1.1 Immediate family support (such as children and spouses)	5.00 ± 0.00	0	0.461
2.1.2 Collateral family support (such as brothers and sisters)	4.87 ± 0.35	0.072	0.305
2.1.3 Friend support	4.53 ± 0.64	0.141	0.122
2.1.4 Original workplace support	4.47 ± 0.64	0.143	0.112
2.2 Social security	4.67 ± 0.49	0.105	0.126
2.2.1 Medical insurance	4.87 ± 0.35	0.072	0.400
2.2.2 Endowment insurance	4.80 ± 0.41	0.086	0.400
2.2.3 Social welfare (such as older adult care service subsidies and nursing subsidies)	4.67 ± 0.48	0.105	0.200
2.3 Public health services	4.60 ± 0.63	0.137	0.111
2.3.1 Management of older adult health and integrated medical care services	4.87 ± 0.35	0.072	0.221
2.3.2 Health literacy promotion project	4.80 ± 0.56	0.117	0.194
2.3.3 Resident health records	4.60 ± 0.74	0.160	0.085
2.3.4 Community Health Education	4.60 ± 0.74	0.160	0.085
2.3.5 Designated hospitals for follow-up visits	4.87 ± 0.35	0.072	0.221
2.3.6 Older adult welfare home / older adult care institution services	4.80 ± 0.56	0.117	0.194
2.4 Family function	4.93 ± 0.26	0.052	0.349
2.4.1 Family caregivers	4.87 ± 0.35	0.072	0.333
2.4.2 Family communication	4.93 ± 0.26	0.052	0.333
2.4.3 Family cohesion	4.87 ± 0.35	0.072	0.333
2.5 Economic level	4.60 ± 0.51	0.110	0.111
2.5.1 Personal fixed annual income	4.73 ± 0.59	0.125	0.387
2.5.2 Personal investment income	4.53 ± 0.74	0.164	0.170
2.5.3 Child support income	4.80 ± 0.41	0.086	0.443
2.6 Living environment	4.60 ± 0.74	0.160	0.111
2.6.1 Public activities and entertainment venues	4.60 ± 0.51	0.110	0.040
2.6.2 Home environment	5.00 ± 0.00	0	0.286
2.6.3 Auxiliary walking tools	5.00 ± 0.00	0	0.286
2.6.4 Emergency alarm instrument	5.00 ± 0.00	0	0.143
2.6.5 High and low drop warning	4.80 ± 0.56	0.117	0.120
2.6.6 Public transportation	4.73 ± 0.46	0.097	0.334
2.6.7 Private transportation	4.40 ± 0.63	0.144	0.142
2.6.8 Traffic safety facilities	4.87 ± 0.35	0.072	0.525
3 Individual-environment interaction	5.00 ± 0.00	0	0.429
3.1 Social participation	4.67 ± 0.49	0.105	1.000
3.1.1 Income-based work	4.53 ± 0.64	0.141	0.540
3.1.2 Housework	4.20 ± 0.68	0.161	0.164
3.1.3 Social activities	4.40 ± 0.74	0.167	0.297
3.2 Personal motivation and willingness	4.27 ± 0.80	0.187	0.052
3.2.1 Health behavior awareness	4.53 ± 0.83	0.184	0.667
3.2.2 Self value pursuit	4.20 ± 1.21	0.287	0.333

### Weight of indicators

The Saaty Scale was determined based on the mean value difference of the importance score of each indicator given by experts in the second round of expert consultation, and a judgment matrix was constructed for each indicator level. A hierarchical construction model diagram was established using yaahp 12.9 statistical software, to obtain the weight of each level indicator. The weight of the first-level indicator is 0.143–0.429. The weight of second-level indicators is 0.052–0.349; The weight of and third-level indicator is 0.040–0.667, as shown in Table 4.

## Discussion

This study considered the functional ability in older adults, integrating stroke-specific health status evaluation to develop this evaluation index system. Three core elements–intrinsic capacity, external environment, and individual-environment interactions are highlighted in the indicator. Therefore, the evaluation index system was comprehensive. The expert panel selection adhered to strict inclusion criteria, with 15 of 20 experts, completing two rounds of consultations. The effective recovery rates of the two rounds of the Delphi survey questionnaires were 90 and 83.3%, respectively, indicating that experts were enthusiastic about this study and met the analysis requirements of the expert inquiry results (22). The proportion of experts who proposed opinions in the two rounds was 61.1 and 40%, respectively, indicating that experts pay more attention to the evaluation of functional ability of older patients with stroke, so the study results have good content validity. The Cr values of the two rounds of experts were 0.84 and 0.86, respectively, indicating that experts have a greater degree of confidence in the evaluation of indicators, and the representativeness and authority of the experts are good, so the results of expert consultation are relatively reliable. Kendall’s W values for the two rounds of expert consultation were 0.238–0.278 and 0.122–0.333 (*p* < 0.05). Moreover, Kendall’s W value of the second round of expert consultation was higher than that of the first round, indicating that after the second round of expert consultation, the experts’ opinions were consistent and the study results were relatively stable. The analytic hierarchy process yielded the weights of various evaluation indicators by constructing a judgment matrix and conducting consistency checks on the judgment matrix. The CR value for each indicator level was <0.1, indicating that the indicator weights are accurate and reasonable.

The WHO’s reconceptualization of healthy aging may facilitate a shift from disease-centered to function-centered (23). Most older patients with stroke may experience varying degrees of dysfunction, including physical and mental disabilities and social maladaptation, which can lead to varying degrees of impairment in stroke patients’ intrinsic capacities (24, 25), resulting in patients not being able to interact well with the surrounding environment. Thus, patients cannot maintain their functional abilities, which makes it difficult for them to achieve their true health. Therefore, most healthcare professionals are currently paying more attention to implementing targeted interventions for older patients with stroke, such as limb rehabilitation training, psychological remodeling, and family and social support. However, practical changes must be based on precise evaluation. This study developed an evaluation index system for the functional ability of older patients with stroke based on the framework of healthy aging, highlighting the concept that the essence of health is functional ability (26), fully reflecting the key role of intrinsic capacity, environmental factors, and the interaction between the two in improving functional ability in older patients with stroke. The evaluation index system is clear and easy to understand and can provide a theoretical basis for the development of functional ability assessment tools for older patients with stroke to evaluate their functional ability of older patients with stroke in a multidimensional and targeted manner; it provides a reference for medical and healthcare personnel to implement precise interventions and has profound significance in promoting patient health and improving their quality of life.

Among the first-level indicators, “intrinsic capacity” had the highest weight, and the coefficient of variation was 0, indicating that experts unanimously believed that intrinsic capacity plays a decisive role in the functional ability levels of older patients with stroke. Intrinsic capacity reflects the overall state of older individuals, including their mobility, physical function, cognitive ability, and psychological status. In addition, the ICF (17) released by the World Health Organization in 2001 is considered the origin of the development of the concept of intrinsic capacity. Compared with the impact of diseases, intrinsic capacity focuses more on the various functions that older individuals need to complete what they consider important. However, the need for functional abilities may vary among older individuals patients with stroke. For example, patients without physical disabilities may wish to participate in social activities, whereas stroke patients with disabilities may believe that being able to live independently fulfills their function. In this study, the weight of “external environment” and “individual-environment interaction” are 0.143 and 0.429, indicating that when environmental factors combine with intrinsic capacities to form a mutual relationship, the functional ability of patients can be determined. Compared with the individual role of intrinsic capacity, the environment can usually help individuals achieve better functional ability, and a suitable environment can promote functional ability by eliminating certain obstacles to intrinsic capacity.

The weights of “activity ability” and “psychological state,” “family function” and “social support,” as well as social participation, respectively, rank in the top two among the secondary-level indicators, indicating that only by ensuring that patient’s physiological, psychological, family and social adaptation are in a good state can they achieve a high level of functional ability, that is, achieve healthy aging, which is consistent with the concept of health (27). Unlike previous studies (23), we believe that the motivation and willingness of older patients with stroke are prerequisites for their functioning based on the above explanation of the connotation of functional ability. Therefore, we added “personal motivation and willingness” to the second-level indicators corresponding to intrinsic capacity, because individual motivation is an important internal driving force that activates individual behavior. The higher the level of individual motivation, the stronger the subjective willingness to execute that behavior. Additionally, this study categorized social participation as an interaction between individuals and the environment, which may differ from the findings of previous studies. Most studies identify social participation as a social factor affecting the health of older individuals (28, 29). However, social participation refers to the conscious and behavioral participation of individuals in various aspects of society, such as the economy, politics, culture, and community work. This reflects the role played by individuals or groups in social activities, as well as their interactions with other members of society (30). Therefore, the connotation of social participation is not limited to an individual’s abilities or surrounding social environmental factors, but rather the result of the interaction between the individual’s social ability and the surrounding environment.

In this study, all third-level indicators were determined based on the conceptual connotations of secondary-level indicators and the characteristics of the diseases in older patients with stroke. For “activity function,” we include both ADLs and IADLs, which are good indicators for evaluating the functional ability of older patients with stroke, as they reflect the intrinsic capability and the external support of patients. The “sensory function” proposed by WHO includes vision and hearing ability. In this study, considering that taste, smell, and deep and shallow sensory abilities of the body may also be impaired in patients with stroke, we added these three indicators. However, these sensory impairments do not exist in isolation and often synergistically affect physical and cognitive functions, thereby affecting the overall quality of life (31, 32). Therefore, it is necessary to comprehensively evaluate patients’ sensory abilities. Regarding the “psychological state,” we included “Post-stroke comorbid anxiety and depression” (PSCAD) based on the psychological characteristics of stroke patients. Epidemiological surveys show that the prevalence of PSCAD is approximately 27.81–40.69% (33, 34), of which belong to the category of post-stroke emotional disorders, and the two often occur successively, exacerbating each other. Vitality refers to the dietary intake of energy required to maintain the optimal balance level of the body. Energy consumption and metabolism are important components in the process of mourning and aging and are important indicators for measuring the physical condition of older individuals (35). Therefore, we determined two indicators including “nutritional status” and “sleep status.” In addition, considering the impact of the disease and complications in stroke patients, they may experience physical pain, which, in turn, affects their vitality. Therefore, we have added the indicator of “chronic pain.” Meanwhile, experts believe that the vascular health status of stroke patients plays an important role in ensuring their vitality; therefore, we adopted expert opinions. In terms of “social support,” we further refined the support of relatives, dividing it into “immediate family support” and “collateral family support.” At the same time, we learned during the interview that the patient’s workplace before the illness would provide certain economic support; therefore, we added the indicator “original workplace support.” For “public health services,” we focused more on the specific content of the services. In addition to some health promotion projects currently carried out in communities and older adult care institutions, we have added the indicator of “designated hospitals for follow-up visits” to evaluate the follow-up of stroke patients in the later stage. Regarding the “living environment,” as most stroke patients have certain physical and mental disabilities, the indicators we include pay more attention to evaluating the safety, convenience, entertainment, and surrounding transportation conditions of the patient’s living environment.

### Limitations

Owing to study funding and time constraints, the expert panel was restricted to 15 experts from different regions of China; some experts were not interviewed in the second round of the Delphi survey, which may have led to some bias in the results, potentially limiting the generalizability of expert opinions. Additionally, the evaluation index system has not yet been applied, and the evaluation of the functional ability of older patients with stroke based on the evaluation index system needs to be further determined. The accessibility of the evaluation data, the operability of the indicators, and the empirical stability and reasonableness of the weights of the indicators must be further verified.

## Conclusion

The study employed the modified Delphi method to develop a functional ability evaluation index system for older patients with stroke, comparing three first-level indicators, 13 s-level indicators, and 53 third-level indicators, and determined the weight of each indicator using scientific and application values. This index system incorporates the WHO theoretical model of healthy aging, highlighting the combinations of intrinsic capacity, external environment, and individual-environmental interactions, which can provide a reference for medical staff to evaluate the functional ability of older patients with stroke. Future research should focus on developing and validating a comprehensive assessment tool based on these evaluation indicators to determine their clinical ability.

## Data Availability

The raw data supporting the conclusions of this article will be made available by the authors, without undue reservation.

## References

[1] United Nations, Department of Economic and Social Affairs, population division, world population prospects 2022. (2022) Available online at: https://population.un.org/wpp/ (Accessed 7 November, 2024).

[2] Tu WJ Zeng XW Liu Q. Aging tsunami coming: the main finding from China’s seventh national population census. Aging Clin Exp Res. (2022) 34:1159–63. doi: 10.1007/s40520-021-02017-4, PMID: 34727357

[3] Su YJ CT CH Liang C Lee PF Lin CF Chen HT. Association between health-related physical fitness and self-reported health status in older Taiwanese adults. BMC Geriatr. (2022) 22:235. doi: 10.1186/s12877-022-02929-4, PMID: 35313813 PMC8939111

[4] Calderón-Larrañaga A Vetrano DL Onder G Gimeno-Feliu LA Coscollar-Santaliestra C Carfí A. Assessing and measuring chronic multimorbidity in the older population: a proposal for its operationalization. J Gerontol A Biol Sci Med Sci. (2017) 72:glw233–glw1423. doi: 10.1093/gerona/glw233, PMID: 28003375 PMC5861938

[5] Wang YN Wu SM Liu M. Temporal trends and characteristics of stroke in China in the past 15 years. West China Med J. (2021) 36:803–7. doi: 10.7507/1002-0179.202105046

[6] GBD 2016 Neurology Collaborators. Global, regional, and national burden of neurological disorders, 1990-2016: a systematic analysis for the global burden of disease study 2016. Lancet Neurol. (2019) 18:459–80. doi: 10.1016/S1474-4422(18)30499-X 30879893 PMC6459001

[7] Siegel JS Shulman GL Corbetta M. Mapping correlated neurological deficits after stroke to distributed brain networks. Brain Struct Funct. (2022) 227:3173–87. doi: 10.1007/s00429-022-02525-7, PMID: 35881254 PMC12057035

[8] Husseini NE Katzan IL Rost NS. Margaret Lehman Blake Cognitive impairment after ischemic and hemorrhagic stroke: a scientific statement from the American Heart Association/American stroke association. Stroke. (2023) 54:e272–91. doi: 10.1161/STR.0000000000000430, PMID: 37125534 PMC12723706

[9] Medeiros GC Roy D Kontos N Beach SR. Post-stroke depression: a 2020 updated review. Gen Hosp Psychiatry. (2020) 66:70–80. doi: 10.1016/j.genhosppsych.2020.06.011, PMID: 32717644

[10] World Health Organization. World report on ageing and health. Geneva: World Health Organization (2015).

[11] Beard JR Officer A de Carvalho IA Sadana R Pot AM Michel JP. The world report on ageing and health: a policy framework for healthy ageing. Lancet. (2016) 387:2145–54. doi: 10.1016/S0140-6736(15)00516-4, PMID: 26520231 PMC4848186

[12] Cesari M de Carvalho IA Thiyagarajan JA Cooper C Martin FC Reginster JY. Evidence for the domains supporting the construct of intrinsic capacity. J Gerontol A Biol Sci Med Sci. (2018) 73:1653–60. doi: 10.1093/gerona/gly011, PMID: 29408961

[13] Jones CA Colletti CM Ding MC. Post-stroke dysphagia: recent insights and unanswered questions. Curr Neurol Neurosci Rep. (2020) 20:61. doi: 10.1007/s11910-020-01081-z, PMID: 33136216 PMC7604228

[14] Miyahara S Maeda K Kawamura K Matsui Y Satake S Arai H. Association between intrinsic capacity and oral health in older patients in a frailty clinic. Eur Geriatr Med. (2024) 15:1119–27. doi: 10.1007/s41999-024-00956-5, PMID: 38438830

[15] Guo J Wang J Sun W Liu X. The advances of post-stroke depression: 2021 update. J Neurol. (2022) 269:1236–49. doi: 10.1007/s00415-021-10597-4, PMID: 34052887

[16] Jünger S Payne SA Brine J Radbruch L Brearley SG. Guidance on conducting and REporting DElphi studies (CREDES) in palliative care: recommendations based on a methodological systematic review. Palliat Med. (2017) 31:684–706. doi: 10.1177/0269216317690685, PMID: 28190381

[17] World Health Organization. International classification of functioning, disability and health: ICF. Geneva: World Health Organization (2001).

[18] Lp H. SAS statistical analysis course [M]. Beijing, China: Beijing, China Electronic Industry Press (2010).

[19] Babbie E. The practice of social research (10th e) Farmington Mound, Michigan, USA: Thompson Wadsworth (2007).

[20] Zeng G Hui L. SAS statistical analysis course. Beijing, China: Pecking Union Medical College Union Press (1994).

[21] Cheng C Liu Y Wang R. The test for Kendall’s coefficient of concordance W conducted by SPSS. J Taishan Med Col. (2010) 31:487–90. doi: 10.3969/j.issn.1004-7115.2010.07.002

[22] Heuzenroeder L Ibrahim F Khadka J Woodman R Kitson A. A Delphi study to identify content for a new questionnaire based on the 10 principles of dignity in care. J Clin Nurs. (2022) 31:1960–71. doi: 10.1111/jocn.15462, PMID: 32799400

[23] Belloni G Cesari M. Frailty and intrinsic capacity: two distinct but related constructs. Front Med. (2019) 6:133. doi: 10.3389/fmed.2019.00133, PMID: 31275941 PMC6591451

[24] Zawadzka E Domańska L. Emotional and social characteristics of stroke patients with low verbal memory. Aging Clin Exp Res. (2018) 30:1203–10. doi: 10.1007/s40520-018-0894-0, PMID: 29340965

[25] Kristensen MG Busk H Wienecke T. Neuromuscular electrical stimulation improves activities of daily living post stroke: a systematic review and Meta-analysis. Arch Rehabil Res Clin Transl. (2021) 4:100167. doi: 10.1016/j.arrct.2021.100167, PMID: 35282150 PMC8904887

[26] Beard JR Si YF Liu ZX Chenoweth L Hanewald K. Intrinsic capacity: validation of a new WHO concept for healthy aging in a longitudinal Chinese study. J Gerontol A Biol Sci Med Sci. (2022) 77:94–100. doi: 10.1093/gerona/glab226, PMID: 34343305

[27] van Druten VP Bartels EA van de Mheen D de Vries E Kerckhoffs APM Nahar-van Venrooij LMW. Concepts of health in different contexts: a scoping review. BMC Health Serv Res. (2022) 22:389. doi: 10.1186/s12913-022-07702-2, PMID: 35331223 PMC8953139

[28] Sowa A Tobiasz-Adamczyk B Topór-Mądry R Poscia A la Milia DI. Predictors of healthy ageing: public health policy targets. BMC Health Serv Res. (2016) 16:289. doi: 10.1186/s12913-016-1520-5, PMID: 27609315 PMC5016728

[29] Wu YT Daskalopoulou C Terrera GM Niubo AS Rodríguez-Artalejo F Ayuso-Mateos JL. Education and wealth inequalities in healthy ageing in eight harmonised cohorts in the ATHLOS consortium: a population-based study. Lancet Public Health. (2020) 5:e386–94. doi: 10.1016/S2468-2667(20)30077-3, PMID: 32619540 PMC7739372

[30] Hashidate H Shimada H Fujisawa Y Yatsunami M. An overview of social participation in older adults: concepts and assessments. Phys Ther Res. (2021) 24:85–97. doi: 10.1298/ptr.R0013, PMID: 34532203 PMC8419478

[31] Li ZZ Tang Z Wang R. Status of elderly disability in 7 cities of China. Chin J Epidemiol. (2016) 37:1561–4. doi: 10.3760/cma.j.issn.0254-6450.2016.12.001 27998398

[32] Pi HY Li Y. Analysis on quality of life and influencing factors among the disabled elderly in Beijing. Chin J Mod Nurs. (2016) 22:193–7. doi: 10.3760/ema.j.issn.1674-2907.2016.02.012

[33] Liegey JS Sagnier S Debruxelles S Poli M Olindo S Renou P. Influence of inflammatory status in the acute phase of stroke on post-stroke depression. Rev Neurol. (2021) 177:941–6. doi: 10.1016/j.neurol.2020.11.005, PMID: 33610348

[34] Williams OA Demeyere N. Association of Depression and Anxiety with Cognitive Impairment 6 months after stroke. Neurology. (2021) 96:e1966–74. doi: 10.1212/WNL.0000000000011748, PMID: 33632803

[35] Escala A. Universal relation for life-span energy consumption in living organisms: insights for the origin of aging. Sci Rep. (2022) 12:2407. doi: 10.1038/s41598-022-06390-6, PMID: 35190571 PMC8861023

