# Supplementary material for: Development of evaluation index system for functional ability of older patients with stroke based on healthy aging: a modified Delphi study
Source: Front Public Health. 2025 Mar 13;13:1562429. doi: 10.3389/fpubh.2025.1562429 (PMC11966419; doi:10.3389/fpubh.2025.1562429)
Supplement: Supplementary file 1 [file Table_1.DOCX]

Supplemental file 1：Search Strategy

**PubMed** **Search Strategy**

1. "Aged"[Mesh] OR “elder” [Title/Abstract] OR “elders” [Title/Abstract] OR “elderly” [Title/Abstract] OR “elder*” [Title/Abstract] OR “old” [Title/Abstract] OR “older people” [Title/Abstract] OR “old age” [Title/Abstract] OR “older adult” [Title/Abstract] OR “older adults” [Title/Abstract] OR “older*” [Title/Abstract] OR “geriatrics” [Title/Abstract]

2. “stroke" [MeSH] OR "cerebrovascular disorders" [MeSH] OR "brain ischemia" [MeSH] OR cerebral infarction*" [MeSH] OR "stroke" [Title/Abstract] OR "apoplexy" [Title/Abstract] OR "cerebrovascular accident" [Title/Abstract] OR "CVA" [Title/Abstract] OR "brain vascular accident" [Title/Abstract] OR "acute stroke" [Title/Abstract] OR "cerebral ischemia" [Title/Abstract] OR "brain ischemia" [Title/Abstract] OR "cerebral infarction" [Title/Abstract] OR "brain infaretion" [Title/Abstract] OR "intracranial hemorrhages" [Title/Abstract ] OR "poststroke" [Title/Abstract] OR "poststroke" [Title/ Abstract ] OR "brain diseases" [Title/Abstract] OR "cerebrovascular disorders" [Title/Abstract]

3. "Healthy Aging" [Mesh] OR “healthy ageing” [Title/Abstract] OR “Aging” [Title/Abstract] OR “Ageing” [Title/Abstract] OR “Ageing well” [Title/Abstract]

4. “Functional ability” [Title/Abstract] OR “Functional abilities” [Title/Abstract] OR “Functional capacity” [Title/Abstract] OR “Functional capacities” [Title/Abstract] OR “Intrinsic capacity” [Title/Abstract] OR “Intrinsic capacities” [Title/Abstract]

5. 1 AND 2 AND 3 AND 4

6. Limited: Publication date from database’s inception - May 2023

**CINAHL Search Strategy**

1. TI ("Aged" OR "elder" OR "elder*"OR "old" OR "old*" OR "older*" OR "geriatrics")

2. TI (“stroke” OR "cerebrovascular disorders" OR "brain ischemia" OR “cerebral infarction” OR "apoplexy" OR "cerebral ischemia" OR “cerebral hemorrhages”)

3. TI ("Healthy Aging" OR "Aging" OR "Ageing well")

4. AB ("Functional abilit*" OR "Functional capacit*" OR "intrinsic capacit*")

5. (1 AND 2 AND 3 AND 4)

6. Limited: Publication date: database’s inception - May 2023

**PsycINFO Search Strategy**

1. TI ("Aged" OR "elder" OR "elder*"OR "old" OR "old*" OR "older*"OR "geriatrics" )

2. TI (“stroke” OR "cerebrovascular disorders" OR "brain ischemia" OR “cerebral infarction” OR "apoplexy" OR "cerebral ischemia" OR “cerebral hemorrhages”)

3. TI ("Healthy Aging" OR "Aging" OR "Ageing well")

4. AB ("Functional abilit*" OR" Functional capacit*" OR "Intrinsic capacit*")

5. (1 AND 2 AND 3 AND 4)

6. Limited: Publication date: database’s inception - May 2023

**Elsevier ScienceDirect Search Strategy**

1. Title, abstract, keywords ("Aged" OR "elder" OR "old" OR "geriatrics" )
2. Title, abstract, keywords (“stroke” OR "cerebrovascular disorders" OR "brain ischemia" OR “cerebral infarction” OR "apoplexy" OR "cerebral ischemia" OR “cerebral hemorrhages”)
3. Title, abstract, keywords ("Healthy Aging" OR "Aging" OR "Ageing well")
4. Title, abstract, keywords ("Functional ability" OR "Functional capacity" OR "Intrinsic capacity")
5. (1 AND 2 AND 3 AND 4)

5. Limited: Publication date: database’s inception - May 2023

**Wiley Online Library Search Strategy**

1. ("Aged" OR "elder" OR "elderly" OR "elder*" OR "old" OR "older" OR "old age" OR "older adult" OR "older*" OR "geriatrics" ) in Abstract
2. (“stroke” OR "cerebrovascular disorders" OR "brain ischemia" OR “cerebral infarction” OR "apoplexy" OR "cerebral ischemia" OR “cerebral hemorrhages”) in Title
3. ("Healthy Aging" OR "Healthy ageing" OR "Aging" OR "Ageing" OR "Ageing well") in Abstract
4. ("Functional capacity" OR "Functional capacities" OR "Intrinsic capacity" OR "Intrinsic capacities") anywhere

5. (1 AND 2 AND 3 AND 4)

6. Limited: Publication date: database’s inception - May 2023

**China National Knowledge Infrastructure (CNKI) Search Strategy**

1. 主题=老年 OR 主题=老年人
2. 主题=脑卒中 OR 主题=卒中 OR 主题=中风 OR 主题=脑梗死 OR 主题=脑出血
3. 主题=健康OR 主题=健康老龄化
4. 主题=功能OR 主题=功能发挥OR 主题=功能水平OR 主题=内在能力
5. 1 AND 2 AND 3 AND 4
6. Limited: Publication date: database’s inception - May 2023

**Wan Fang databases(万方数据库) Search Strategy**

1. 主题=老年 OR 主题=老年人
2. 主题=脑卒中 OR 主题=卒中 OR 主题=中风 OR 主题=脑梗死 OR 主题=脑出血
3. 主题=健康OR 主题=健康老龄化
4. 主题=功能OR 主题=功能发挥OR 主题=功能水平OR 主题=内在能力
5. 题名或关键词=老年 OR 题名或关键词=老年人
6. 题名或关键词=脑卒中 OR 题名或关键词=卒中 OR 题名或关键词=中风 OR 题名或关键词=脑梗死 OR 题名或关键词=脑出血
7. 题名或关键词=健康OR 题名或关键词=健康老龄化
8. 题名或关键词=功能OR 题名或关键词=功能发挥OR 题名或关键词=功能水平OR 题名或关键词=内在能力

9. (1 AND 2 AND 3 AND 4) OR (5 AND 6 AND 7 AND 8)

10. Limited: Publication date: database’s inception - May 2023

**VIP Search (维普网) Strategy**

1. 主题=老年 OR 主题=老年人
2. 主题=脑卒中 OR 主题=卒中 OR 主题=中风 OR 主题=脑梗死 OR 主题=脑出血
3. 主题=健康OR 主题=健康老龄化
4. 主题=功能 OR 主题=功能发挥OR 主题=功能水平OR 主题=内在能力
5. 1 AND 2 AND 3 AND 4
6. Limited: Publication date: database’s inception - May 2023
