# Supplementary material for: Development of evaluation index system for functional ability of older patients with stroke based on healthy aging: a modified Delphi study
Source: Front Public Health. 2025 Mar 13;13:1562429. doi: 10.3389/fpubh.2025.1562429 (PMC11966419; doi:10.3389/fpubh.2025.1562429)
Supplement: Supplementary file 2 [file Table_2.DOCX]

Supplemental file 2: Interview guide

| Categories | Interview questions |
| --- | --- |
| Body functions | 1.How do you feel your body is recovering? |
|  | 2.What other problems do you think you have physically and mentally? Can you be more specific with me? |
| Body structures | 3.What parts of your body do you feel are defective or damaged? |
|  | 4.How do you feel your physical condition affects you? Can you be more specific? |
| Activities and participation | 5.What activities do you usually do? Can you talk about an example? |
|  | 6.What are any problems with your daily activities? Can you be more specific with me? |
| Environmental factors | 7.Which of your surroundings and living conditions do you feel are favourable for your recovery? Can you give me some examples? |
|  | 8. What are the disadvantages of your surroundings and living conditions? |
