# Supplementary material for: Development of evaluation index system for functional ability of older patients with stroke based on healthy aging: a modified Delphi study
Source: Front Public Health. 2025 Mar 13;13:1562429. doi: 10.3389/fpubh.2025.1562429 (PMC11966419; doi:10.3389/fpubh.2025.1562429)
Supplement: Supplementary file 3 [file Table_3.DOCX]

Supplemental file 3: Expert inquiry questionnaire

**Expert inquiry questionnaire (The first round)**

Instructions for filling out the form:

This survey is only for academic study, and we will strictly keep your personal information and the information you provide confidential. Please fill in your personal information or check the appropriate options with a "√".

Table 1 Questionnaire of expert basic information

| Name |  | Gender |  | Age |  | Education |  |
| --- | --- | --- | --- | --- | --- | --- | --- |
| Professional title |  | Position |  | Study field |  | Years of service |  |
| Work unit |  | | | | | | |
| Graduate Tutor | □Master's Supervisor □Doctoral supervisor □No | | | | | | |
| Telephone |  | | | E-mail |  | | |

Instructions for filling out the form:

Use the Likert 5-point method to classify the importance of each indicator. Please assess the importance of each indicator and check the appropriate options with a "√" (single choice) in the corresponding column. Welcome to add your suggestions in the "Modification suggestions" and "Additional items needed" columns. The indicators you added also need to be judged for the importance.

Table 2 First level indicator evaluation form

| First level indicators | Concept | Delete | Modification suggestions | Degree of importance | | | | |
| --- | --- | --- | --- | --- | --- | --- | --- | --- |
|  |  |  |  | Unimportant（1） | less important（2） | General（3） | Important（4） | very important（5） |
| Intrinsic capacity | The integration of individual physiological and psychological abilities based on genetics and influenced by personal and health characteristics |  |  |  |  |  |  |  |
| External environment | All external factors that make up the living background of the elderly, including the micro environment composed of family environment, living environment, and interpersonal relationships, as well as the macro environment of public policies and social interactions |  |  |  |  |  |  |  |
| Individual-environment interaction | The dynamic development and interactive nature of the relationship between individuals and the environment |  |  |  |  |  |  |  |
| Additional items needed | |  | |  |  |  |  |  |

Table 3 Sencond level indicator evaluation form

| First level indicators | Sencond level indicators | Delete | Modification suggestions | Degree of importance | | | | |
| --- | --- | --- | --- | --- | --- | --- | --- | --- |
|  |  |  |  | Unimportant（1） | less important（2） | General（3） | Important（4） | very important（5） |
| Intrinsic capacity | Activity function |  |  |  |  |  |  |  |
|  | Sensory function |  |  |  |  |  |  |  |
|  | Cognitive function |  |  |  |  |  |  |  |
|  | Psychological state |  |  |  |  |  |  |  |
|  | Vitality state |  |  |  |  |  |  |  |
|  | Additional items needed |  | |  |  |  |  |  |
| External environment | Interpersonal relationship |  |  |  |  |  |  |  |
|  | Social support |  |  |  |  |  |  |  |
|  | Social security |  |  |  |  |  |  |  |
|  | Public health services |  |  |  |  |  |  |  |
|  | Family environment |  |  |  |  |  |  |  |
|  | Economic level |  |  |  |  |  |  |  |
|  | Living area |  |  |  |  |  |  |  |
|  | Living environment |  |  |  |  |  |  |  |
|  | Housing environment |  |  |  |  |  |  |  |
|  | Traffic conditions |  |  |  |  |  |  |  |
|  | Public security environment |  |  |  |  |  |  |  |
|  | Additional items needed |  | |  |  |  |  |  |
| Individual-environment interaction | Social participation |  |  |  |  |  |  |  |
|  | Additional items needed |  | |  |  |  |  |  |

Table 4 Third level indicator evaluation form

| Sencond level indicators | Third level indicators | Delete | Modification suggestions | Degree of importance | | | | |
| --- | --- | --- | --- | --- | --- | --- | --- | --- |
|  |  |  |  | Unimportant（1） | less important（2） | General（3） | Important（4） | very important（5） |
| Activity function | Basic daily activity ability |  |  |  |  |  |  |  |
|  | Instrumental daily activity ability |  |  |  |  |  |  |  |
|  | motor functional ability |  |  |  |  |  |  |  |
|  | Additional items needed |  | |  |  |  |  |  |
| Sensory function | Visual ability |  |  |  |  |  |  |  |
|  | Hearing ability |  |  |  |  |  |  |  |
|  | Hemianesthesia |  |  |  |  |  |  |  |
|  | Gustatory ability |  |  |  |  |  |  |  |
|  | Olfactory ability |  |  |  |  |  |  |  |
|  | Additional items needed |  | |  |  |  |  |  |
| Cognitive function | Memory |  |  |  |  |  |  |  |
|  | Attention |  |  |  |  |  |  |  |
|  | Calculation ability |  |  |  |  |  |  |  |
|  | Directive force |  |  |  |  |  |  |  |
|  | Execution ability |  |  |  |  |  |  |  |
|  | Language expression |  |  |  |  |  |  |  |
|  | Communication skills |  |  |  |  |  |  |  |
|  | Additional items needed |  | |  |  |  |  |  |
| Psychological state | Post-stroke comorbid anxiety and depression |  |  |  |  |  |  |  |
|  | Positive emotions |  |  |  |  |  |  |  |
|  | Psychological resilience |  |  |  |  |  |  |  |
|  | Self-efficacy |  |  |  |  |  |  |  |
|  | Additional items needed |  | |  |  |  |  |  |
| Vitality state | Nutritional state |  |  |  |  |  |  |  |
|  | Sleep state |  |  |  |  |  |  |  |
|  | mental state |  |  |  |  |  |  |  |
|  | Additional items needed |  | |  |  |  |  |  |
| Interpersonal relationship | Kinship |  |  |  |  |  |  |  |
|  | Neighborhood relations |  |  |  |  |  |  |  |
|  | Friends and relationships with others |  |  |  |  |  |  |  |
|  | Additional items needed |  | |  |  |  |  |  |
| Social support | Child support |  |  |  |  |  |  |  |
|  | Family support |  |  |  |  |  |  |  |
|  | Friend support |  |  |  |  |  |  |  |
|  | Additional items needed |  | |  |  |  |  |  |
| Social security | Medical insurance |  |  |  |  |  |  |  |
|  | Endowment insurance |  |  |  |  |  |  |  |
|  | Social welfare |  |  |  |  |  |  |  |
|  | Social mutual assistance |  |  |  |  |  |  |  |
|  | Additional items needed |  | |  |  |  |  |  |
| Public health services | Resident health records |  |  |  |  |  |  |  |
|  | Health education |  |  |  |  |  |  |  |
|  | Elderly rehabilitation hospital |  |  |  |  |  |  |  |
|  | Nursing home |  |  |  |  |  |  |  |
|  | Additional items needed |  |  |  |  |  |  |  |
| Family environment | Family members |  |  |  |  |  |  |  |
|  | Emotional expression |  |  |  |  |  |  |  |
|  | Family cohesion |  |  |  |  |  |  |  |
|  | Additional items needed |  |  |  |  |  |  |  |
| Economic level | Personal annual income |  |  |  |  |  |  |  |
|  | Real estate |  |  |  |  |  |  |  |
|  | Additional items needed |  | |  |  |  |  |  |
| Living area | City |  |  |  |  |  |  |  |
|  | Village |  |  |  |  |  |  |  |
|  | countryside |  |  |  |  |  |  |  |
|  | Additional items needed |  | |  |  |  |  |  |
| Living environment | Community medical services |  |  |  |  |  |  |  |
|  | Public activities and entertainment venues |  |  |  |  |  |  |  |
|  | Community ageing facilities |  |  |  |  |  |  |  |
|  | Elderly cultural centers |  |  |  |  |  |  |  |
|  | Additional items needed |  |  |  |  |  |  |  |
| Housing environment | Housing quality |  |  |  |  |  |  |  |
|  | Smartphones |  |  |  |  |  |  |  |
|  | Additional items needed |  |  |  |  |  |  |  |
| Traffic conditions | Transportation facilities |  |  |  |  |  |  |  |
|  | Traffic safety |  |  |  |  |  |  |  |
|  | Additional items needed |  | |  |  |  |  |  |
| Public security environment | Community Safety |  |  |  |  |  |  |  |
|  | Additional items needed |  | |  |  |  |  |  |
| Social participation | Participation in volunteer activities |  |  |  |  |  |  |  |
|  | Income-based work |  |  |  |  |  |  |  |
|  | Housework |  |  |  |  |  |  |  |
|  | Social activities |  |  |  |  |  |  |  |
|  | Participation in elderly clubs |  |  |  |  |  |  |  |
|  | Additional items needed |  | |  |  |  |  |  |

Instructions for filling out the form:

The following is the judgment basis and familiarity of expert. Please mark "√" in the corresponding column.

Table 5 Judgement basis and familiarity of expert

| Judgement basis | Large | Medium | Small |
| --- | --- | --- | --- |
| Practical experience |  |  |  |
| Theoretical analysis |  |  |  |
| Understanding of peers |  |  |  |
| Intuitive choice |  |  |  |
| Familiarity | □very familiar □more familiar □generally familiar □less familiar □unfamiliar | | |

**Expert inquiry questionnaire (The second round)**

Instructions for filling out the form:

This survey is only for academic study, and we will strictly keep your personal information and the information you provide confidential. Please fill in your personal information or check the appropriate options with a "√".

Table 1 Questionnaire of expert basic information

| Name |  | Gender |  | Age |  | Education |  |
| --- | --- | --- | --- | --- | --- | --- | --- |
| Professional title |  | Position |  | Study field |  | Years of service |  |
| Work unit |  | | | | | | |
| Graduate Tutor | □Master's Supervisor □Doctoral supervisor □No | | | | | | |
| Telephone |  | | | E-mail |  | | |

Instructions for filling out the form:

Use the Likert 5-point method to classify the importance of each indicator. Please assess the importance of each indicator and check the appropriate options with a "√" (single choice) in the corresponding column. Welcome to add your suggestions in the "Modification suggestions" and "Additional items needed" columns. The indicators you added also need to be judged for the importance.

Table 2 First level indicator evaluation form

| First level indicators | Concept | Delete | Modification suggestions | Degree of importance | | | | |
| --- | --- | --- | --- | --- | --- | --- | --- | --- |
|  |  |  |  | Unimportant（1） | less important（2） | General（3） | Important（4） | very important（5） |
| Intrinsic capacity | The integration of individual physiological and psychological abilities based on genetics and influenced by personal and health characteristics |  |  |  |  |  |  |  |
| External environment | All external factors that make up the living background of the elderly, including the micro environment composed of family environment, living environment, and interpersonal relationships, as well as the macro environment of public policies and social interactions |  |  |  |  |  |  |  |
| Individual-environment interaction | The dynamic development and interactive nature of the relationship between individuals and the environment |  |  |  |  |  |  |  |
| Additional items needed | |  | |  |  |  |  |  |

Table 3 First level indicator evaluation form

| First level indicators | Sencond level indicators | Delete | Modification suggestions | Degree of importance | | | | |
| --- | --- | --- | --- | --- | --- | --- | --- | --- |
|  |  |  |  | Unimportant（1） | less important（2） | General（3） | Important（4） | very important（5） |
| Intrinsic capacity | Activity function |  |  |  |  |  |  |  |
|  | Sensory function |  |  |  |  |  |  |  |
|  | Cognitive function |  |  |  |  |  |  |  |
|  | Psychological state |  |  |  |  |  |  |  |
|  | Vitality state |  |  |  |  |  |  |  |
|  | Additional items needed |  | |  |  |  |  |  |
| External environment | Social support |  |  |  |  |  |  |  |
|  | Social security |  |  |  |  |  |  |  |
|  | Public health services |  |  |  |  |  |  |  |
|  | Family function |  |  |  |  |  |  |  |
|  | Economic level |  |  |  |  |  |  |  |
|  | Living environment |  |  |  |  |  |  |  |
|  | Additional items needed |  | |  |  |  |  |  |
| Individual-environment interaction | Social participation |  |  |  |  |  |  |  |
|  | Personal motivation and willingness |  |  |  |  |  |  |  |
|  | Additional items needed |  | |  |  |  |  |  |

Table 4 Third level indicator evaluation form

| Sencond level indicators | Third level indicators | Delete | Modification suggestions | Degree of importance | | | | |
| --- | --- | --- | --- | --- | --- | --- | --- | --- |
|  |  |  |  | Unimportant（1） | less important（2） | General（3） | Important（4） | very important（5） |
| Activity function | Basic daily activity ability |  |  |  |  |  |  |  |
|  | Instrumental daily activity ability |  |  |  |  |  |  |  |
|  | Additional items needed |  | |  |  |  |  |  |
| Sensory function | Visual ability |  |  |  |  |  |  |  |
|  | Hearing ability |  |  |  |  |  |  |  |
|  | Deep and shallow sensory ability of the body |  |  |  |  |  |  |  |
|  | Gustatory ability |  |  |  |  |  |  |  |
|  | Olfactory ability |  |  |  |  |  |  |  |
|  | Additional items needed |  | |  |  |  |  |  |
| Cognitive function | Memory |  |  |  |  |  |  |  |
|  | Attention |  |  |  |  |  |  |  |
|  | Calculation ability |  |  |  |  |  |  |  |
|  | Directive force |  |  |  |  |  |  |  |
|  | Language ability |  |  |  |  |  |  |  |
|  | Visual spatial discrimination ability |  |  |  |  |  |  |  |
|  | Additional items needed |  | |  |  |  |  |  |
| Psychological state | Post-stroke comorbid anxiety and depression |  |  |  |  |  |  |  |
|  | Positive emotions |  |  |  |  |  |  |  |
|  | Psychological resilience |  |  |  |  |  |  |  |
|  | Self-efficacy |  |  |  |  |  |  |  |
|  | Additional items needed |  | |  |  |  |  |  |
| Vitality state | Nutritional state |  |  |  |  |  |  |  |
|  | Sleep state |  |  |  |  |  |  |  |
|  | Chronic pain |  |  |  |  |  |  |  |
|  | Vascular health state |  |  |  |  |  |  |  |
|  | Additional items needed |  | |  |  |  |  |  |
| Social support | Child support |  |  |  |  |  |  |  |
|  | Family support |  |  |  |  |  |  |  |
|  | Friend support |  |  |  |  |  |  |  |
|  | Original workplace support |  |  |  |  |  |  |  |
|  | Additional items needed |  | |  |  |  |  |  |
| Social security | Medical insurance |  |  |  |  |  |  |  |
|  | Endowment insurance |  |  |  |  |  |  |  |
|  | Social welfare (such as elderly care service subsidies and nursing subsidies) |  |  |  |  |  |  |  |
|  | Additional items needed |  | |  |  |  |  |  |
| Public health services | Management of elderly health and integrated medical care services |  |  |  |  |  |  |  |
|  | Health literacy promotion project |  |  |  |  |  |  |  |
|  | Resident health records |  |  |  |  |  |  |  |
|  | Community Health Education |  |  |  |  |  |  |  |
|  | Designated hospitals for follow-up visits |  |  |  |  |  |  |  |
|  | Elderly welfare home / elderly care institution services |  |  |  |  |  |  |  |
|  | Additional items needed |  |  |  |  |  |  |  |
| Family function | Family caregivers |  |  |  |  |  |  |  |
|  | Family communication |  |  |  |  |  |  |  |
|  | Family cohesion |  |  |  |  |  |  |  |
|  | Marital status |  |  |  |  |  |  |  |
|  | Additional items needed |  | |  |  |  |  |  |
| Economic level | Personal fixed annual income |  |  |  |  |  |  |  |
|  | Personal investment income |  |  |  |  |  |  |  |
|  | Additional items needed |  |  |  |  |  |  |  |
| Living environment | Public activities and entertainment venues |  |  |  |  |  |  |  |
|  | Home environment (such as floors, indoor lighting, floor anti slip, safety handrails, bathing and defecating assistance facilities, and home interior cleaning and beautification) |  |  |  |  |  |  |  |
|  | Auxiliary walking tools |  |  |  |  |  |  |  |
|  | Emergency alarm instrument |  |  |  |  |  |  |  |
|  | High and low drop warning |  |  |  |  |  |  |  |
|  | Public transportation |  |  |  |  |  |  |  |
|  | Private transportation |  |  |  |  |  |  |  |
|  | Traffic safety facilities |  |  |  |  |  |  |  |
|  | Additional items needed |  | |  |  |  |  |  |
| Social participation | Income-based work |  |  |  |  |  |  |  |
|  | Housework |  |  |  |  |  |  |  |
|  | Social activities |  |  |  |  |  |  |  |
|  | Additional items needed |  | |  |  |  |  |  |
| Personal motivation and willingness | Health behavior awareness |  | |  |  |  |  |  |
|  | Self value pursuit |  | |  |  |  |  |  |
|  | Additional items needed |  |  |  |  |  |  |  |

Instructions for filling out the form:

The following is the judgment basis and familiarity of expert. Please mark "√" in the corresponding column.

Table 5 Judgement basis and familiarity of expert

| Judgement basis | Large | Medium | Small |
| --- | --- | --- | --- |
| Practical experience |  |  |  |
| Theoretical analysis |  |  |  |
| Understanding of peers |  |  |  |
| Intuitive choice |  |  |  |
| Familiarity | □very familiar □more familiar □generally familiar □less familiar □unfamiliar | | |

**Judgement basis and the degree of influence**

| Judgement basis | Degree of influence | | |
| --- | --- | --- | --- |
|  | Samll (0) | Medium (0.5) | Large (1) |
| Practical experience (0.4) | 0 | 0.2 | 0.4 |
| Theoretical analysis (0.3) | 0 | 0.15 | 0.3 |
| Understanding of peers (0.2) | 0 | 0.1 | 0.2 |
| Intuitive choice (0.1) | 0 | 0.05 | 0.1 |
| Total | 0 | 0.5 | 1 |
