# Supplementary material for: Development of evaluation index system for functional ability of older patients with stroke based on healthy aging: a modified Delphi study
Source: Front Public Health. 2025 Mar 13;13:1562429. doi: 10.3389/fpubh.2025.1562429 (PMC11966419; doi:10.3389/fpubh.2025.1562429)
Supplement: Supplementary file 4 [file Table_4.DOCX]

Supplemental file 4: Content of indicator revision

**First round of indicator revision**

| Indicators | Add | Merge (before) | Merge (after) | Modify (before) | Modify (after) | Exclude |
| --- | --- | --- | --- | --- | --- | --- |
| Second-level Indicators | Self-discipline ability | Interpersonal relationships | Social support | Family environment | Family function | Living area |
|  |  | Social support |  |  |  |  |
|  |  | Housing environment | Living environment |  |  | Public security environment |
|  |  | Transport condition |  |  |  |  |
| Third-level Indicators | Visual spatial discrimination ability | / | / | Hemianesthesia | Deep and shallow sensory ability of the body | Motor functional ability |
|  | Chronic pain |  |  | language expression | language ability | Execution ability |
|  | Vascular health status |  |  | Social welfare | Social welfare (such as elderly care service subsidies and nursing subsidies) | Mental state |
|  | Original unit support |  |  | Health education | Community health education | Participation in volunteer activities |
|  |  |  |  | Family members | Family caregivers | Participation in elderly clubs |
|  | Management of elderly health and integrated medical care services |  |  | Emotional expression | Family communication | Social mutual assistance |
|  |  |  |  | Personal annual income | Personal fixed annual income | Community ageing facilities |
|  | Health literacy promotion project |  |  | Traffic safety | Traffic safety facilities | Elderly cultural centers |
|  |  |  |  | Transportation facilities | Public transportation | Housing quality |
|  | Personal investment income |  |  |  | Private transportation | Smartphones |

**Second round of indicator revision**

| Indicators | Add | Modify (before) | Modify (after) | Exclude |
| --- | --- | --- | --- | --- |
| Second-level Indicators | Personal motivation and willingness | / | / | Self-discipline ability |
|  |  |  |  |  |
|  |  |  |  |  |
| Third-level Indicators | Child support income | Child support | Immediate family support (such as children and spouses) | Marital status |
|  | Health behavior awareness |  |  |  |
|  | Self value pursuit | Family support | Collateral family support (such as brothers and sisters) | Communication skills |
|  | Home environment (such as floors, indoor lighting, floor anti slip, safety handrails, bathing and defecating assistance facilities, and home interior cleaning and beautification) | Elderly rehabilitation hospital | Designated hospital services | Real estate |
|  | Auxiliary walking tools |  |  |  |
|  | Emergency alarm instrument | Nursing home | Elderly welfare home / elderly care institution services | Community medical services |
|  | High and low drop warning |  |  |  |
